# Biogeographical Evidences Help Revealing the Origin of Hainan Island

**DOI:** 10.1371/journal.pone.0151941

**Published:** 2016-04-07

**Authors:** Hua Zhu

**Affiliations:** Center for Integrative Conservation, Xishuangbanna Tropical Botanical Garden, Chinese Academy of Sciences, Kunming, Yunnan, P. R. China; Chinese Academy of Forestry, CHINA

## Abstract

Both the continental or island origin of Hainan, and the Indo-Malaysian or East Asian affinity of its flora, are debatable. In this study, 196 families, 1283 genera and 3894 species of Hainan plants were recognized. Patterns of seed plant distribution were quantified at the generic and the family levels. The floras of Vietnam, and the adjacent Guangxi and Guangdong provinces of mainland China, were compared with Hainan. The results showed that the flora of Hainan Island is dominated by families and genera with tropical distributions. Among its floristic elements, tropical Asian distributions make up 23.85% of the total flora, while East Asian distributions contribute only 3.74%. There are only 7 genera and c. 10% of species endemic to Hainan Island. The Hainan flora has strong similarities to the flora of Vietnam at the family and the generic levels, and also to the flora of Guangxi, but less with the adjacent Guangdong province. The main conclusions are: Hainan’s flora is tropical in nature with a strongly tropical Asian affinity, and it has very low endemism at the generic and species levels, which implies a continental origin. We conjecture that Hainan Island could have been adjacent to northern Vietnam and the Guangxi at least in Eocene. This suggestion is supported by palaeobotanical, palaeomagnetic and volcanism studies.

## Introduction

Hainan Island, a province of southern China, lies at 108°36′43″–111°2′31″ E and 18°10′04″–20°9′40″ N, with an area of 33920 km^2^. It has high mountains in the centre and lowlands at the periphery. Hainan Island has a typical tropical climate with the annual mean temperate of 23–25°C. The island has a relatively wet climate in the east, with 2000–2400 mm annual rainfall, and a dry climate in the west, with only 1000–1200 mm. November to the May is the dry season, and the end of May to October is the rainy season, with 70–90% of the annual rainfall.

Hainan Island was originally covered by tropical rain forest, tropical monsoon forest and savanna in the lowlands, and tropical montane forest in the highlands [1]. Hainan’s flora has been studied by several different authors [2, 3, 4, 5]. The latest complete record of the flora of Hainan shows that it is composed of 3715 species of native seed plants in 1102 genera and 218 families [6]. Only 397 endemic species have been recognized [7], which is low compared with other tropical islands. The low levels of endemism of the flora of Hainan could reflect its continental nature, but it has a highly disharmonic flora when compared with that from mainland China [8]. The Hainan flora was traditionally considered to be a part of Chinese mainland flora [3, 4], however its Indo-Malaysia affinity was suggested [9, 10, 11, 12].

Palaeobotanical studies indicate that Hainan had a subtropical flora and a climate in the Eocene [13, 14, 15], which was very different from the present. The geological history of Hainan is debatable. Hainan Island is only 20 km from the mainland at the narrowest point after the formation of the Qiongzhou strait. A common idea is that it was previously connected to Guangdong on the mainland and that the Qiongzhou strait was formed in the middle Holocene when rising sea levels flooded the low-lying land between them [16, 17]. However, a totally different idea is that the Qiongzhou strait was formed by an active rift structure in the early Pleistocene [18, 19, 20], and that Hainan Island was originally located near Guangxi and northern Vietnam during the early Cenozoic, as suggested by palaeomagnetic evidence [21]. From the late Mesozoic to early Cenozoic, the Beibu Gulf lithosphere was drawn away, and Hainan moved southeast along Red River fault and revolved clockwise to the present location. Therefore, the continental or “island” origin of Hainan, and the Indo-Malaysian or mainland China affinity of its flora, are still debatable.

Understanding the affinity and evolution of the Hainan flora is important for understanding the plant geography of Hainan Island and its origin. This paper aims to (i) analyze the floristic patterns and geographical elements of Hainan; (ii) examine the affinity and evolution of the Hainan flora; (iii) provide biogeographical evidence for the origin of the island.

## Materials and Methods

Based mainly on the inventory of Hainan plants [6], I made a revision on the native plant species by checking, synonymizing, and adding species from our database, and 196 families, 1283 genera and 3894 species of native seed plants were recognized in Hainan (S1 Appendix). The circumscriptions of families follow APG III [22, 23] and species nomenclature follows w^3^TROPICOS (http://mobot.mobot.org/W3T/Search/vast.html). Patterns of seed plant distribution were quantified at the generic and the family levels following Wu [24] and Wu et al. [25, 26] as: Cosmopolitan, Pantropic, Tropical Asia and Tropical America disjunct, Old World Tropics, Tropical Asia to Tropical Australia, Tropical Asia to Tropical Africa, Tropical Asia, North Temperate, East Asia and North America disjunct, Old World Temperate, Temperate Asia, Mediterranean region, West to Central Asia, Central Asia, East Asia and Endemic to China. The biogeographical affinity of the flora was studied using geographical elements at the family and generic levels. The floras of three neighboring regions, Vietnam [27, 28], Guangxi Province [29] and Guangdong Province [30] were selected for comparison in order to understand their relationships. The geological history, and the palaeomagnetic and palaeobotanical evidence from Hainan, are also discussed.

## Results

### Floristic composition

A total of 3894 native seed plant species from 1283 genera and 196 families were recognized from Hainan Island. There were 9 families with more than 100 species including Poaceae (267 species), Orchidaceae (264), Fabaceae (230), Rubiaceae (199), Cyperaceae (172), Euphorbiaceae (160), Asteraceae (125), Lamiaceae (110), and Lauraceae (103). There were 6 families with 51–100 species, i.e., Asclepiadaceae, Acanthaceae, Fagaceae, Moraceae, Annonaceae and Convolvulaceae (Table 1). These 15 most species-rich families, with a total of 1993 species, contribute 51.2% of the total flora, although they make up only 7.7% of the total families. In contrast, there were 122 families with 1–10 species, which make up 62.2% of the total families, but contribute only 11.4% of the total flora. As is usual, the most species-rich families have worldwide distributions. However, the families Euphorbiaceae and Lauraceae, as well as most dominant families with 30–100 species, have pantropic distributions.

**Table 1:** Dominant families and genera in species richness with their distribution.

Family ranking by their species richness	No. of species in the flora	Distribution type*	Genera ranking by their species richness	No. of species in the flora	Distribution type*
Poaceae	267	1	*Ficus*	41	2
Orchidaceae	264	1	*Hedyotis*	40	2
Fabaceae	230	1	*Syzygium*	36	4
Rubiaceae	199	1	*Ilex*	36	2
Cyperaceae	172	1	*Fimbristylis*	35	2
Euphorbiaceae	160	2	*Cyperus*	31	1
Asteraceae	125	1	*Ardisia*	29	2
Lamiaceae	110	1	*Carex*	29	1
Lauraceae	103	2	*Lithocarpus*	25	9
Asclepiadaceae	68	2	*Lasianthus*	25	2
Acanthaceae	66	2	*Symplocos*	25	2
Fagaceae	64	8	*Bambusa*	22	5
Moraceae	60	1	*Polygonum*	22	1
Annonaceae	54	2	*Cyclobalanopsis*	21	7
Convolvulaceae	51	1	*Dendrobium*	20	5
Myrsinaceae	49	2	*Bulbophyllum*	19	2
Urticaceae	49	2	*Diospyros*	19	2
Myrtaceae	48	2	*Callicarpa*	18	2
Scrophulariaceae	48	1	*Smilax*	18	2
Rutaceae	42	2	*Liparis*	18	1
Apocynaceae	41	2	*Desmodium*	17	9
Theaceae	41	2	*Litsea*	17	3
Vitaceae	41	2	*Crotalaria*	17	2
Melastomataceae	39	2	*Solanum*	17	1
Araceae	38	2	*Elaeocarpus*	16	5
Aquifoliaceae	36	3	*Beilschmiedia*	16	2
Oleaceae	34	1	*Eragrostis*	16	2
Zingiberaceae	33	5	*Piper*	16	2
Celastraceae	32	2	*Alpinia*	15	7
Rosaceae	31	1	*Camellia*	15	7

*Distribution type: 1: cosmopolitan, 2: pantropic, 3: tropical Asia and tropical America disjunct, 4 Old World Tropic, 5 Tropical Asia to Tropical Australia, 6 Tropical Asia to Tropical Africa, 7 Tropical Asia, 8 North Temperate, 9 East Asia and North America disjunct

The most species-rich genus is Ficus with 41 species, following by the genera Hedyotis (40), Syzygium (36) and Ilex (36). There are 15 genera with more than 20 species and 50 genera with 11–20 species (Table 1). A majority of these species-rich genera have pantropic distributions, such as Ficus, Hedyotis, Ilex, Fimbristylis and Ardisia. These species-rich genera include 1119 species, making up 5% of the total genera, and contributing 28.7% of the total species. There are 1124 genera with 1–5 species, which include 2075 species, contributing 53.3% of the number of species, but forming 87.6% of the total number of genera.

### Geographical elements

Of the 196 families, 123 families are tropical elements (types 2–7), contributing 62.6% of the flora (Table 2). Among these, 84 families (or 42.9%) have pantropic distributions, such as Acanthaceae, Anacardiaceae, Annonaceae, Apocynaceae, Euphorbiaceae, Lauraceae, Melastomataceae and Rutaceae; 13 families (6.6%) have tropical Asian and tropical American disjunct distributions, such as Aquifoliaceae, Araliaceae, Gesneriaceae, Elaeocarpaceae, Styracaceae. Families with old world tropic distributions make up 4.6%, and families with tropical Asia to tropical Australia distributions and tropical Asia distributions each make up 3.6%.

**Table 2:** Geographical elements of seed plants at the family level in the flora of Hainan Island.

Geographical elements at family level	Number of family	%*
1 Cosmopolitan	45	23.0
2 Pantropic	84	42.9
3 Tropical Asia and Tropical America disjunct	13	6.6
4 Old World Tropic	9	4.6
5 Tropical Asia to Tropical Australia	7	3.6
6 Tropical Asia to Tropical Africa	3	1.5
7 Tropical Asia	7	3.6
Tropical elements (types 2–7) in total	123	62.8
8 North Temperate	20	10.2
9 East Asia and North America disjunct	6	3.1
10 East Asia	2	1.0
Temperate elements (types 8–10) in total	28	14.3
Total number of families	196	100

*The number of families in each geographical element/ the number of families of all geographical elements times 100.

Temperate elements (types 8–10) include 28 families, contributing 14.3% of the flora. Among them, 20 families (10.2%) have north temperate distributions, including Caprifoliaceae, Cornaceae, Fagaceae, and Hamamelidaceae; 6 families have east Asia and north America disjunct distributions, such as Magnoliaceae, Saururaceae, Schisandraceae, contributing 3.1%; 2 families, Cephalotaxaceae and Actinidiaceae, have east Asia distributions.

Fifteen distribution types at generic level were recognized from the flora (Table 3). Of the 1283 genera, tropical elements (types 2–7) contribute 80.5%, and temperate elements (types 8–14) contribute 14.3%. Among tropical genera, tropical Asian distributions have the highest proportion with 305 genera, contributing 23.9% of the total flora. Pantropic distributions with 296 genera, contribute 23.1%. Other tropical genera have tropical Asia to tropical Australia distributions, which make up 13.2% and old world tropic distributions, which make up 11.5%. Among temperate genera, 4.6% have northern temperate distribution and 3.7% have east Asian distributions. Evidently, the Hainan flora is a tropical in nature and is characterized by tropical Asian and pantropic distributions.

**Table 3:** Geographical elements of seed plants at the generic level in the flora of Hainan Island.

Geographical elements at generic level	Number of genera	%*
1 Cosmopolitan	65	5.1
2 Pantropic	296	23.1
3 Tropical Asia and Tropical America disjunct	37	2.9
4 Old World Tropic	147	11.5
5 Tropical Asia to Tropical Australia	169	13.2
6 Tropical Asia to Tropical Africa	80	6.2
7 Tropical Asia	305	23.8
Tropical elements (types 2–7) in total	1034	80.5
8 North Temperate	59	4.6
9 East Asia and North America disjunct	32	2.5
10 Old World Temperate	20	1.6
11 Temperate Asia	4	0.3
12 Mediterranean, W Asia to C Asia	2	0.2
13 East Asia	48	3.7
14 Endemic to China	19	1.5
Temperate elements (types 8–14) in total	184	14.3
Total number of genera	1283	100.0

*The number of genera in each geographical element/ the number of genera of all geographical elements times 100.

There are 19 genera that are endemic or near-endemic to China, including *Oligostachyum* Z. P. Wang & G. H. Ye, *Ampelocalamus* S. L. Chen, T. H. Wen & G. Y. Sheng, *Chieniodendron* Tsiang & P. T. Li, *Cyclocarya* Iljinskaya, *Didymostigma* W. T. Wang, *Metapetrocosmea* W. T. Wang, *Parakmeria* Hu & W. C. Cheng, *Semiliquidambar* H. T. Chang, *Sinobambusa* Makino ex Nakai, *Tetrapanax* (K. Koch) K. Koch. Among them are 7 Hainan endemic genera: *Pyrenocarpa* Chang & Miau, *Cathayanthe* Chun, *Chunia* H. T. Chang, *Hainanecio* Y.Liu & Q.R.Yang (Asteraceae), *Pentastelma* Tsiang & P. T. Li, *Setiacis* S. L. Chen & Y. X. Jin, and *Wenchengia* C. Y. Wu & S. Chow.

Genera with high species richness (>10 species) predominantly have pantropic distributions (50.0% in the genera with >20 species, and 52.0% in the genera with 11–20 species) (Table 4). Among genera with 6–10 species, those with pantropic distributions still contribute the highest proportion (34.7%), followed by those with old world tropic distributions (20.0%). Genera with 1–5 species contribute 95.8% of the Hainan flora (1124 genera). They have diverse distributions; those with tropical Asia distributions have the highest proportion and make up 25.9%, followed by pantropic (20.5%), tropical Asia to tropical Australia (13.4%), and old world tropic distributions (10.9%). All 19 Chinese endemic genera included in the Hainan flora have 1–5 species.

**Table 4:** Geographical elements across genera in the flora.

Distribution type	No. of genera (more than 20 species)	%	No. of genera (11–20 species)	%	No. of genera (6–10 species)	%	No. of genera (1–5 species)	%
1 Cosmopolitan	3	21.4	4	8.0	8	8.4	50	4.5
2 Pantropic	7	50.0	26	52.0	33	34.7	230	20.5
3 Tropical Asia and Tropical America disjunct	0	0.0	3	6.0	2	2.1	32	2.9
4 Old World Tropic	1	7.1	4	8.0	19	20.0	123	10.9
5 Tropical Asia to Tropical Australia	1	7.1	7	14.0	10	10.5	151	13.4
6 Tropical Asia to Tropical Africa	0	0.0	0	0.0	5	5.3	75	6.7
7 Tropical Asia	1	7.1	5	10.0	9	9.5	290	25.9
8 North Temperate	0	0.0	0	0.0	6	6.3	53	4.7
9 East Asia and North America disjunct	1	7.1	1	2.0	2	2.1	28	2.5
10 Old World Temperate	0	0.0	0	0.0	0	0.0	20	1.8
11 Temperate Asia	0	0.0	0	0.0	0	0.0	4	0.4
12 Mediterranean, W Asia to C Asia	0	0.0	0	0.0	0	0.0	2	0.2
13 Center Asia	0	0.0	0	0.0	0	0.0	0	0.0
14 East Asia	0	0.0	0	0.0	1	1.1	47	4.2
15 Endemic to China	0	0.0	0	0.0	0	0.0	19	1.6
Total number of genera	14	100.0	50	100.0	95	100.0	1124	100.0

### Comparison to neighboring regional floras

**Fig 1:**
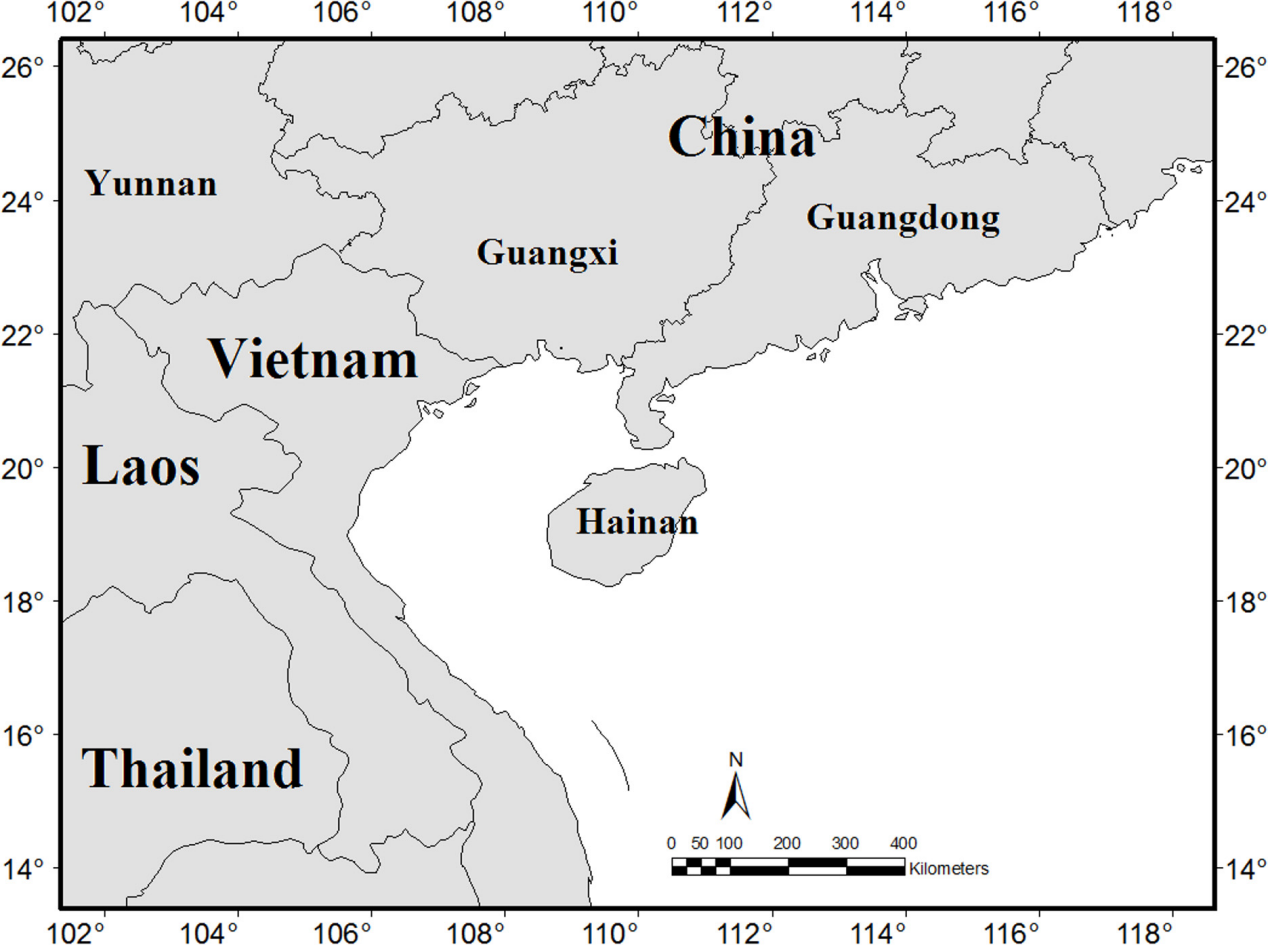
Geographical location of Hainan Island and the compared neighbouring regions (Vietnam, Guangxi and Guangdong of mainland China).

Three neighboring regional floras: Guangxi province to the north-west, Guangdong province to the north-east, and Vietnam to the southern-west, were selected to make comparisons (Fig 1). Guangxi has 209 recorded seed plant families, 1652 genera and 7734 species [29]; Guangdong has 197 families, 1413 genera and 5302 species [30]; and Vietnam has 235 families, 2023 genera and 10790 species (based on Chan [27], checked and synonymized by the author).

Floristic similarities at the family, generic, and species levels between the three neighboring regional floras are detailed in Table 5. The floristic similarities between these regional floras are more than 93.9% at the family level, and more than 78.3% at the generic level, but lower at the species level (35.2–73.1%). However, the flora of Hainan has the highest floristic similarities with that of Vietnam at the family (100%) and generic (89.0%) levels, followed by Guangxi. The Hainan flora is exceptionally related to the floras of Vietnam and Guangxi, although they are farther away than Guangdong. If we look at the genera shared between these regions, we could see that 110 genera are shared by Hainan and Vietnam only, but only 7 genera are shared by Hainan and Guangdong only. Clearly the flora of Hainan is most closely related to the flora of Vietnam.

**Table 5:** Comparison of floristic similarities at the family, generic and specific levels.

Compared flora	Hainan (196 families, 1283 genera and 3894 species)	Vietnam (235 families, 2023 genera and 10790 species)	Guangxi (209 families, 1652 genera and 7734 species)	Guangdong (197 families, 1413 genera and 5302 species)
	Similarity coefficient (%)*	Similarity coefficient (%)	Similarity coefficient (%)	Similarity coefficient (%)
**Similarity coefficients at family level**				
Hainan	100			
Vietnam	100	100		
Guangxi	96.9	97.6	100	
Guangdong	93.9	99.0	99.0	100
**Similarity coefficients at generic level**				
Hainan	100			
Vietnam	89.0	100		
Guangxi	83.2	80.4	100	
Guangdong	78.3	82.7	91.0	100
**Similarity coefficients at specific level**				
Hainan	100			
Vietnam	52.6	100		
Guangxi	61.6	35.2	100	
Guangdong	55.7	39.3	73.1	100

*Similarity coefficient between A and B = the number of taxa shared by both A and B divided by the lowest number of taxa of A or B, multiplied by 100%

## Discussion

### Tropical flora in nature with tropical Asian affinity

The floristic composition of Hainan Island reveals that it is tropical in nature. Of the 196 families in Hainan, tropical elements contribute 62.8% of the flora, while the temperate elements contribute 14.3%. In its 28 temperate families, north temperate distributions make up the majority, and only 2 families have East Asia distributions. At the generic level, tropical elements contribute 80.5%, and temperate elements 14.3%. Furthermore, the tropical Asian distributions make up 23.9% of the total flora, which shows a strongly tropical Asian affinity, while the East Asian distributions contribute only 3.7%.

### Low endemism of the flora of Hainan Island

There are 19 genera that are endemic or approximately endemic to China, including 7 Hainan endemics. There are only 397 endemic species on the island [7], which contribute c.10% of the total flora. The very low endemism of the flora indicates that Hainan Island has a continental origin, compared with other tropical islands. For example, the volcanic-originated Hawaii has a flora with endemic plants making up to 90% of its total flora [31]. A comparable island in tropical Asia is Sri Lanka. Sri Lanka and peninsular India comprise a single tectonic structure known as the Deccan Plate. Geological evidence suggests that Adam's Bridge is a former land connection between India and Sri Lanka. Sri Lanka and India had intermittent lowland connections until recently [32]. There are 171 families including 1056 genera and 2855 angiosperm plants in Sri Lanka, of which 27 genera and 853 species are endemic to the island [33]. Although one quarter of the angiosperm flora of Sri Lanka is endemic to the continental island [34], 942 of its 1056 genera are shared with the Indian peninsula [34], which reveals the land connection with India. Compared with Sri Lanka, Hainan Island has a lower number of endemics, which implies that its isolation from the mainland is more recent. There are 1124 genera with 1–5 species, which contribute 95.8% of the genera of Hainan flora. Except for a small proportion of species-rich genera with pantropic and wide-range distributions, the majority are small genera with low species richness. There are no genera with high endemic richness or an evolution centre in Hainan Island [7].

### Biogeographical similarity to Vietnam and Guangxi

Although Hainan is a continental island, its flora does not have its closest similarity at the family and generic level with the nearest part of the mainland, Guangdong, but with the floras of Vietnam and Guangxi. There are 110 genera shared by Hainan and Vietnam only, but only 7 genera shared by Hainan and Guangdong only.

Among the shared mammals, Hainan also has the highest overlap with Vietnam and the lowest with Guangdong. Among the 41 mammal species in Hainan, there are 30 species also in Vietnam. *Hylobates concolor* Thoms (Hylobatidae) is distributed in Vietnam, Laos and Yunnan, and its subspecies *Hylobates concolor hainanus* occurs in Hainan. Hainan species *Crocidura horsfieldi* Tomes and *C*. *himalayica* Gray are shared with Yunnan and Vietnam; *Tupaia glis modesta* J.Allen (Tupaiidae), *Pygathrix nemaeus* Linnaeus (Cercopithecidae), *Paradoxurus hermaphroditus laotum* Gyodenstolpe (Viverridae), and *Lepus peguensis* Blyth (Leporidae) are shared with Vietnam. Eld’s deer (*Cervus eldi*) (Cervidae) occurs in Hainan and Myanmar at present, and historically in Laos, Vietnam and Thailand [35, 36]. Mammal distributions therefore show a similar pattern to those of angiosperms, with the closest biogeographical relationship with Vietnam.

### The continental origin and southward drift of Hainan Island supported by palaeobotanical, palaeomagnetic and volcanism studies

Palaeobotanical studies suggest that Hainan Island could have been in a much more northern location in the Eocene than at present. A fossil seed cone from the upper Miocene Wenshan of SE Yunnan (23°15′N, 104°15′E, 1482 m a.s.l.) was recognized as *Pinus massoniana* Lambert. The comparisons indicated that the fossils closely resemble *P*. *massoniana* var. *hainanensis*, which is a tropical montane plant restricted to Hainan Island. This implies a close relationship between Hainan and Yunnan [37]. The connection between Yunnan and Hainan was suggested to be through northern Vietnam and/or southwestern Guangxi. Zhao et al. [15] also researched the fossils from the Changchang Basin of Hainan Island and found that the fossil flora was clearly subtropical in the Eocene, with the temperate genera *Abies* and *Tsuga*. The fossil fruits *Palaeocarya* (Juglandaceae) and *Acer* (Sapindaceae) from the Changchang Basin also indicated a relatively cool temperature in the Eocene [38]. *Craigia* (Malvaceae) includes two species: *Craigia kwangsiensis* H.H. Hsue and *Craigia yunnanensis* W.W. Sm. & W.E. Evans. in Guangxi and Yunnan, respectively, but it had fossils in Hainan in the Eocene [39].

The flora of Hainan is tropical now but was more subtropical during the Tertiary. The Cenozoic plant families in Hainan Island were mostly tropical, but there was a high ratio of subtropical to temperate families, which implies a much lower temperature than the present [13] (Jin *et al*., 2008). Yao et al. [14] studied the Eocene palaeoclimate and palaeovegetation in Hainan based on the Changchan fossils and suggested that there was a subtropical evergreen or deciduous broad-leaved forest at the center of the basin, but a temperate evergreen or deciduous broad-leaved forest and needle-leaved forest growing in the peripheral part of the basin, and that the climate in the Changchang Basin during the Early–Middle Eocene was warm and humid subtropical with a mean annual temperature of 14.2°–19.8°C.

**Fig 2:**
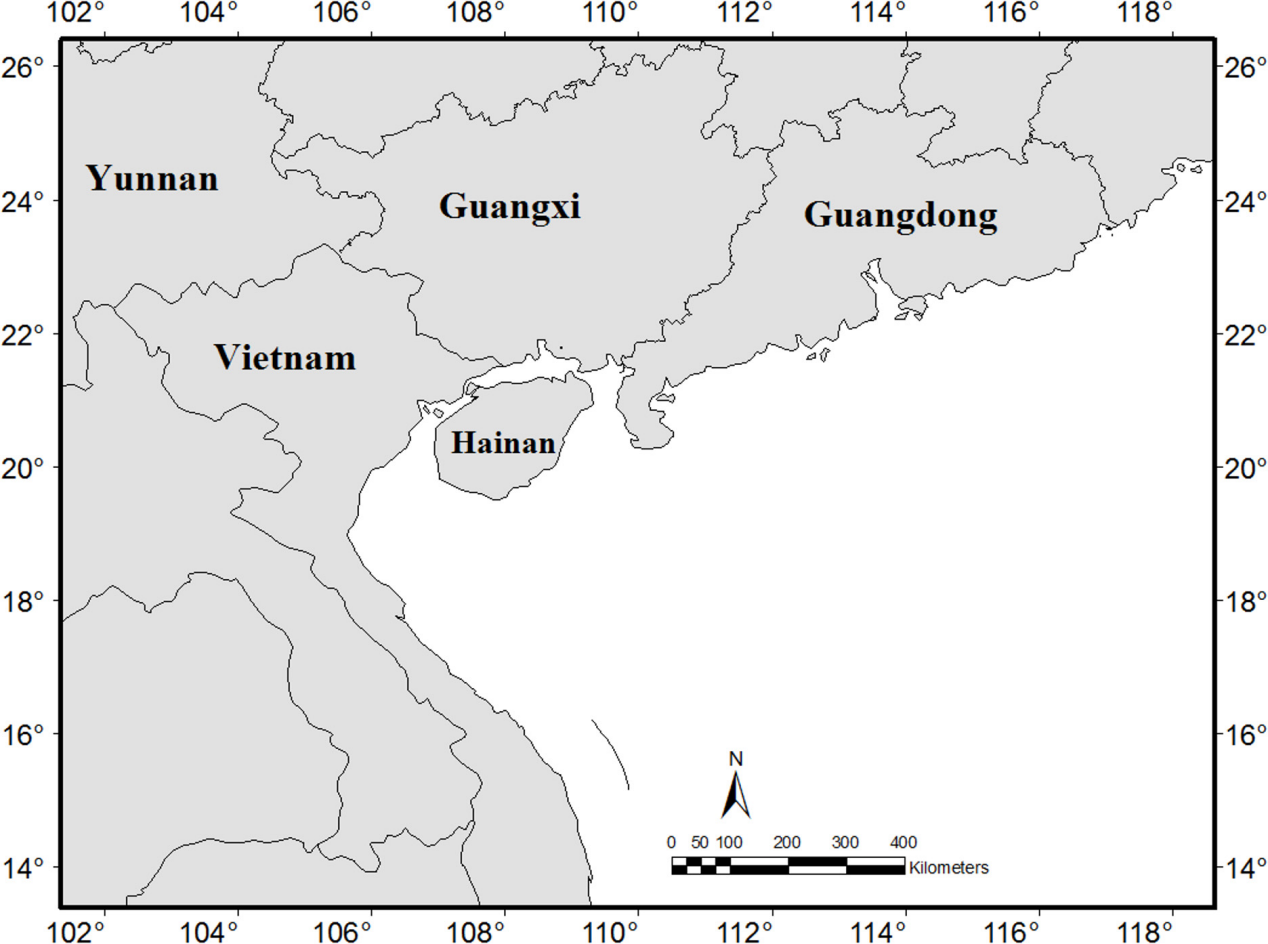
Illustrative the conjecture location of Hainan Island.

Hainan Island is separated from Guangdong by the Qiongzhou strait, which is up to 40 km wide, but only 20 km at the narrowest point, and has a 40–120 m depth [19]. There are large disagreements on the formation of the Qiongzhou strait. It could have been formed by an active rift structure in the early Pleistocene [18, 10, 20], although a different idea was that the strait was formed in the middle Holocene by a marine transgression over the original lowland area [16, 17]. It has been suggested that Hainan Island was previously located near Guangxi and northern Vietnam during the early Cenozoic [21, 40]. This suggestion is strongly supported by our biogeographical studies. Hainan Island has a continental origin, but does not have its closest relation to the adjacent Guangdong province, but to Vietnam and Guangxi (Fig 2).

Palaeomagnetic studies show that Hainan Island was almost connected to north Vietnam and Guangxi during the Mesozoic, at about the location of the Beibu Gulf [21, 41]. Tonkin Gulf had a widespread extension in a 100 km wide zone prior to 30 Ma from the Red River fault system [42]. Evidently, geological evolution of Tonkin-Beibu Gulf caused Hainan Island’s southeast moment. Analysis of rock magnetism and palaeomagnetism shows that Hainan had the paleolatitudes in the Early Cretaceous of 24.8°N and the Late Cretaceous 26.3°N, and was 5–6° north of the present geographic position [43]. The Cretaceous palaeomagnetic results from Hainan Island also indicate that it rotated clockwise by about 25° relative to Indo-Chinese Block in the Early Cretaceous [43]. The Early Cretaceous palaeomagnetic results from Hainan Island demonstrate that Hainan was at 25.9°N and situated at about a 7° higher latitude than the present position (about 19°N) during the Early Cretaceous, and had rotated by 4.0±5.8° and translated southward by 14.1±5.5° relative to the suspected coherent part of the south China block since the Cretaceous [44]. It has been suggested that the rotation of Hainan Island could have occurred during the mid-Tertiary, when large-scale left-lateral motion occurred along the Red River Fault as a result of the indentation of the Indian Plate into Eurasia, causing extrusion of the Indochina Block and the opening of the South China Sea [45].

The Cenozoic volcanism occurred in Leizhou peninsular and northern Hainan shows the characteristics of plate marginal tectonism [46, 47]. This could reflect continental rifting and drifting.

Evidently, palaeomagnetic and volcanism studies reveal that Hainan Island had drifted southward from a more northwestern position. This is supported by our biogeographical studies.

## Conclusion

I can make the following deductions from biogeographical studies in Hainan Island:

Hainan has a tropical flora. Tropical elements contribute 80.5% of the total genera, of which the tropical Asian distributions make up 23.9%, which shows a strongly tropical Asian affinity. Although Hainan is closest to Guangdong, it has only 3.7% of the genera with east Asian distributions. There are only 7 endemic genera and 397 endemic species (contributing to c.10% of the total flora), which indicates that Hainan island has a very low number of endemics and a disharmonic flora. Hainan Island’s flora had a continental origin. Although Hainan Island is only 20 km from Guangdong, it has more floristic similarities to Vietnam and Guangxi at the family and generic levels. There are 110 genera shared by Hainan and Vietnam only, but only 7 shared by Hainan and Guangdong only. Hainan has also the highest proportion of mammals shared with Vietnam, and the lowest proportion with Guangdong. The disharmonic flora of Hainan Island reveals that its present location was not its original site. I deduce that Hainan Island could have been in contact with northern Vietnam and Guangxi at least in Eocene. This would be consistent with the observed biogeography.

Palaeobotanical studies suggest that Hainan Island could have been in a much more northerly location with a subtropical climate in the Eocene. This supports our deduction that Hainan could have been adjacent to Vietnam and Guangxi. Our results support the suggestion from palaeomagnetic and volcanism studies that the Qiongzhou strait could have been formed by an active rift structure, and Hainan drifted to the present location by moving southeast from a higher latitude.

## Supporting Information

S1 Appendix Native seed plant list in Hainan Island. (The circumscriptions of families follow APG II, and species nomenclature follows w^3^TROPICOS (http://mobot.mobot.org/W3T/Search/vast.html).
(XLS)
